# A Flexible Strain Sensor Based on the Porous Structure of a Carbon Black/Carbon Nanotube Conducting Network for Human Motion Detection

**DOI:** 10.3390/s20041154

**Published:** 2020-02-20

**Authors:** Peng Zhang, Yucheng Chen, Yuxia Li, Yao Zhang, Jian Zhang, Liangsong Huang

**Affiliations:** Key Laboratory for Robot Intelligent Technology of Shandong Province, Shandong University of Science and Technology, Qingdao 266590, China; pengzhang@sdust.edu.cn (P.Z.); chenyucheng@sdust.edu.cn (Y.C.); yuxiali2004@sdust.edu.cn (Y.L.); zhangyao@sdust.edu.cn (Y.Z.); zhangjian@sdust.edu.cn (J.Z.)

**Keywords:** flexible strain sensor, carbon black, multi-walled carbon nanotubes, human motion detection

## Abstract

High-performance flexible strain sensors are playing an increasingly important role in wearable electronics, such as human motion detection and health monitoring, with broad application prospects. This study developed a flexible resistance strain sensor with a porous structure composed of carbon black and multi-walled carbon nanotubes. A simple and low-cost spraying method for the surface of a porous polydimethylsiloxane substrate was used to form a layer of synergized conductive networks built by carbon black and multi-walled carbon nanotubes. By combining the advantages of the synergetic effects of mixed carbon black and carbon nanotubes and their porous polydimethylsiloxane structure, the performance of the sensor was improved. The results show that the sensor has a high sensitivity (GF) (up to 61.82), a wide strain range (0%–130%), a good linearity, and a high stability. Based on the excellent performance of the sensor, the flexible strain designed sensor was installed successfully on different joints of the human body, allowing for the monitoring of human movement and human respiratory changes. These results indicate that the sensor has promising potential for applications in human motion monitoring and physiological activity monitoring.

## 1. Introduction

In recent years, various flexible strain sensors which convert mechanically-dependent variables (such as stretching [1], bending [2], and torsion [3]) into electrical signals have been developed. Applications of such sensors include their use as wearable, soft sensor joints on the surface of the skin, which can measure the biological and physiological activities of the user. These advantages and developments have attracted growing attention and applications in artificial intelligence [4,5], human motion detection [6,7,8], human facial expression recognition [9,10], intelligent robots [11,12], and health monitoring [13,14], among others [15]. However, in view of the widespread application of these sensors, several important requirements and considerations need to be met. In order to better monitor the complex signal, the strain sensor should have an adequate flexibility, high sensitivity, large measuring range, fast response ability, excellent durability, and small volume. As well as being comfortable and convenient to wear, easy integration with an external circuit and a low production cost are also key factors that need to be considered. At present, to satisfy the aforementioned characteristics, researchers have designed strain sensors with different sensing mechanisms, among which the resistance sensor has gradually become the mainstream design method of strain sensors due to its simplicity and low cost. According to the response principle of resistance sensors, conductive carbon fillers and mental nanowires (such as carbon black [16,17], carbon nanotubes [18,19], graphene [20,21], and Ag nanowires [22,23]) can be combined with flexible substrates (such as polydimethylsiloxane (PDMS) [24,25], polyurethane (PU) [26,27], silicone rubber (SR) [28,29], elastic fabrics [30,31], and elastic bands [32]) to make a flexible piezoresistive sensor with a high sensitivity and a large stretch range by a certain preparation method. Therefore, these studies reveal that composites made of a conductive material and polymer can meet the performance requirement of strain sensors. In addition, the reasonable fabrication costs and the excellent performances obtained from sensors based on conductive carbon filling materials have made carbon nanotubes and carbon black the best conductive materials for many scholars [33,34].

Carbon black (CB) is a kind of zero-vinami material with a low aspect ratio and large surface area. The conductive channel formed by the contact between carbon black particles can exhibit great changes under low strain levels. However, under higher strain, the gap between carbon black particles increases, which prevents the formation of an effective conductive channel and thus leads to a reduced sensitivity at high levels of strain [35]. As a one-dimensional cylindrical nanostructure, carbon nanotubes (MWNTS) have significant electrical and mechanical properties, and the contact between them can form a close conductive network [36,37]. Due to the light connection between carbon nanotubes, the conductive network formed by carbon nanotubes will not change significantly under low strain, and its resistance only marginally changes. The resistance does not change appreciably until a large strain is applied that destroys the conductive network. Therefore, by combining the characteristics of carbon black and carbon nanotubes, a collaborative conductive network can be established by mixing [34] or layering [38] the two conductive carbon fillers, which not only reduces the production cost, but also significantly improves the electrical characteristics of nano-composite materials. Zheng et al. prepared a strain sensor composed of CB-MWNTs-PDMS mixed in solution, which had a stretching range of up to 300%. Although the sensor could detect human joint movement reasonably well, the monitoring of micro-strain proved to be difficult. Additionally, the large volume of the sensor caused notable discomfort to the user [33]. A layer-by-layer sensor assembly was proposed by Zhang et al., which involved the addition of carbon black and carbon nanotubes to a yard. The sensor was characterized by a simple operation, good linearity, and sensitivity (GF = 45.4), but the conductive material was blocked by Polyvinyl alcohol (PVA) and the synergistic effects of carbon black and carbon nanotubes were not well-exhibited [38].

In this study, in order to improve the sensitivity, reduce the size of the sensor, and increase the wearability, we developed a strain sensor with a high sensitivity, good linear response, large stretch range, and good reliability. Due to its water-soluble properties, granulated sugar was mixed with PDMS to form a stretching matrix with a porous structure after immersion. The experimental data show that the porous structure can significantly improve the stretching range of the sensor to up to 1.3 times its own length. Combined with the unique properties of conductive carbon fillers, such as carbon black and multi-walled carbon nanotubes (MWNTs), a conductive network layer is can be formed on the porous substrate surface through a simple and low-cost spraying method, which improves the sensitivity of the sensor. The flexible strain sensor prepared by this method can monitor the movement of facial muscles and joints, including fingers, wrists, elbows, and knees, as well as other deformations of different magnitudes. In addition, the monitoring of human physiological activities, such as breathing and other minor deformations, can be achieved. These results show that the sensor is a simple, cost-effective solution suitable for large-scale production, with a broad application prospect for wearable monitoring devices. A comparison of the sensor developed in this paper and the characteristics of those proposed in [33] and [38] is shown in Table 1. The variations show evident differences in sensor performance arising from varying methods of preparation.

The specific fabrication method and the process of the flexible strain sensor are discussed in Section 2. Furthermore, Section 3 presents the various sensing characteristics of the flexible strain sensor and analyzes the test results. Finally, a summary and conclusion for the present study are presented in Section 4.

## 2. Design and Fabrication

### 2.1. Experimental Materials

In this experiment, high-purity multi-wall carbon nanotubes (MWNTs) were provided by Nanjing XANANO Materials Tech Co., Ltd., Nanjing, Jiangsu Province, China. The purity of the MWNTs was 95%, with a diameter of 10–20nm, a length of 10–30μm, and a carboxyl content of 2.00 wt%. Carbon black (CB) of the model BP2000 was purchased from Cabot Corporation, Boston, Massachusetts, USA. Alcohol was used as the solvent of conductive carbon fillers, which can accelerate the evaporation of the solvent. The mixture of CB/MWNTs was added to the alcohol for three hours of ultrasonic mixing, and a CB/MWNT dispersion with a mass fraction of 2.00 wt% (1.00 wt% each for CB and MWNTs) was obtained [33]. PDMS was used as the matrix material (Sylgard 184; Dow Corning Corp., Gales Ferry, Connecticut, USA), and the prefabricated mixture was prepared using a 1:15 mass ratio of the curing agent to the substrate, which was stirred for 10 min and set aside. Granulated sugar was ground in the grinder for 30 min for later addition to the matrix.

### 2.2. Fabrication Process of the Porous Flexible Strain Sensor

**Figure 1:**
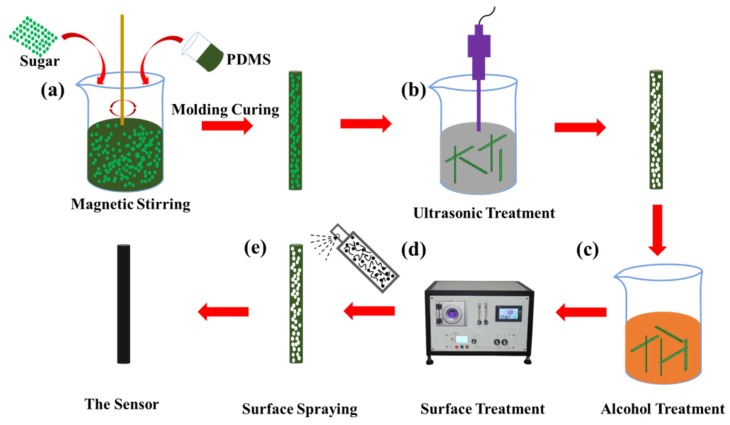
The preparation process of the flexible strain sensor: (**a**) matrix preparation by solution mixing; (**b**) ultrasonic treatment of the matrix in distilled water employed to obtain the porous structure; (**c**) cleaning of the matrix using alcohol; (**d**) the surface of the matrix is treated with isoxic ions to improve the surface activity; (**e**) strain sensors obtained by spraying.

The fabrication process of the porous flexible strain sensor is shown in Figure 1. First, as shown in Figure 1a, the substrate of the porous flexible strain sensor was prepared by the blending method. Mixtures with mass fraction ratios of *m* (sugar)/*m* (PDMS) = *n* (where *n* = 0%, 10%, 20%, 30%, 40% or 50%) were stirred magnetically for 2 h to obtain evenly dispersed sugar/PDMS mixtures. Then, the excess bubbles were removed by vacuum treatment for 20 min, and the various mixtures with different mass fraction ratios were poured into cylindrical molds with an inner diameter of 2.25 mm and cured in an oven at 80 °C for 3h. The substrate of the cured sugar/PDMS mixture was then taken out and placed in distilled water for 48 h of ultrasound treatment to dissolve the granulated sugar within the cured sugar/PDMS mixture completely, and to obtain the porous PDMS substrate, as indicated in Figure 1b. Mechanical tests were conducted on the porous substrate with different mass fraction ratios and the substrate with the best tensile properties was selected (see Section 3.1 for details). The selected substrate was then put into an ethanol solution for 10 min of ultrasonic cleaning to remove any stains and dust on the surface in Figure 1c. After drying, the substrate was rinsed in an oxygen plasma cleaner for 90 s, as shown in Figure 1d. The purpose of this was to improve the surface activity of PDMS by making it hydrophilic and to increase the absorption of conductive nano-materials. Finally, the conductive composite mixture was sprayed evenly on the porous substrate using a spray bottle, and the porous flexible strain sensor was obtained after drying in the oven, as presented in Figure 1e.

### 2.3. Characterization and Electrical Measurements

**Figure 2:**
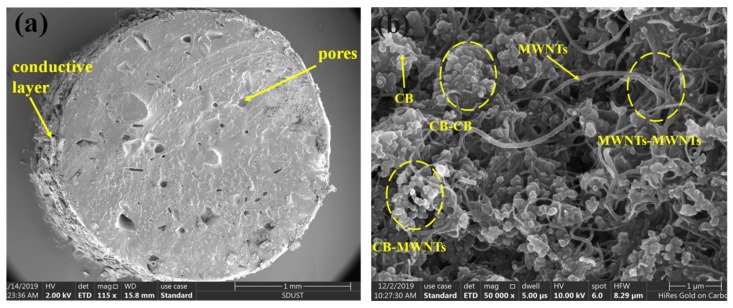
Scanning electron microscopy (SEM) diagram of the flexible strain sensor: (**a**) SEM diagram of the sensor, the porous structure inside the matrix, and the conductive layer on the surface of the matrix and (**b**) SEM image of the conductive network on the sensor surface and the three conductive connection channels (carbon black (CB)-CB, CB-carbon nanotubes (MWNTS), and MWNTS-MWNTS).

The porous flexible strain sensor was characterized by scanning electron microscopy (SEM), and the results are shown in Figure 2. The micrograph of Figure 2a shows evident irregular pores inside the PDMS substrate, indicating that the porous effect was achieved and that a conductive network layer was wrapped around the PDMS substrate. By further magnification of the conductive material coating on the sensor surface, it was revealed that the close contacts of CB and MWNTS crisscross together to form a cooperative conductive network, as exhibited in Figure 2b. The conductive channel was mainly formed by CB-CB, CB-MWNT, and MWNT-MWNT connections (indicated by the yellow circle in Figure 2b), which suggests that the prepared material achieved the desired effect.

**Figure 3:**
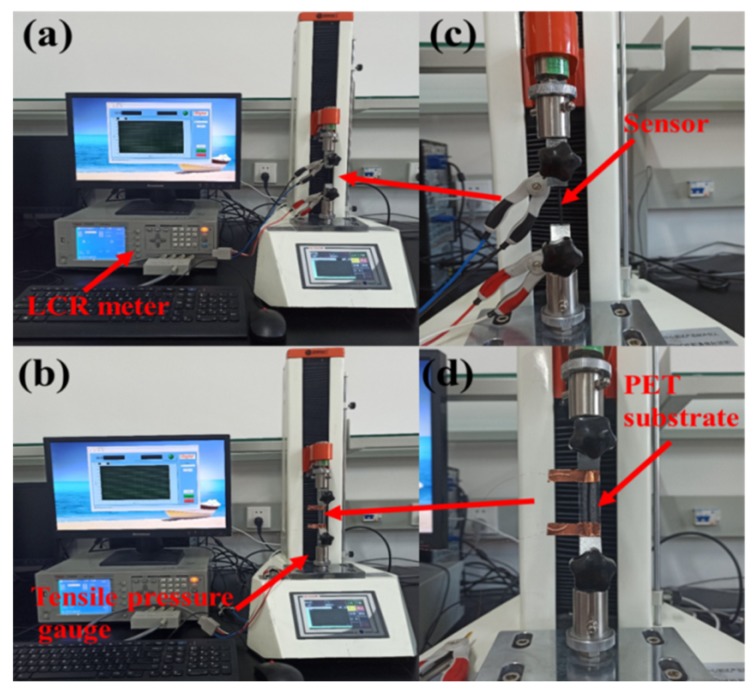
Experimental setup for sensor testing: (**a**) tensile properties testing process diagram; (**b**) bending properties testing process diagram; (**c**) a partially enlarged view of (**a**); (**d**) a partially enlarged view of (**b**).

The experimental setup of the sensor testing process is detailed in Figure 3a,b. By setting the tension gauge to apply strain in different directions, forces were applied to the sensor in both stretching and compressing directions to test the performance parameters of the sensor. The tension gauge was the ZQ-990A model from China Smart Instrument Co., Ltd., with a measuring range of 0~50 N and a force resolution ratio of 0.01 N (Figure 3). The resistance parameter testing instrument was a desktop LCR meter (TH2826) from Changzhou Tonghui Electronics Co., Ltd. As shown in Figure 3a, during the testing of tensile properties, both ends of the strain sensor were fixed firmly to the fixture. During the testing of bending characteristics, the strain sensor was fixed on the PET substrate, which is shown in Figure 3b. A temperature measuring instrument (303-0a, Shanghai kuntian laboratory instrument Co., Ltd.) was used to record the temperature of the sensor.

## 3. Results and Discussion

### 3.1. Characteristics of the Flexible Strain Sensor

**Figure 4:**
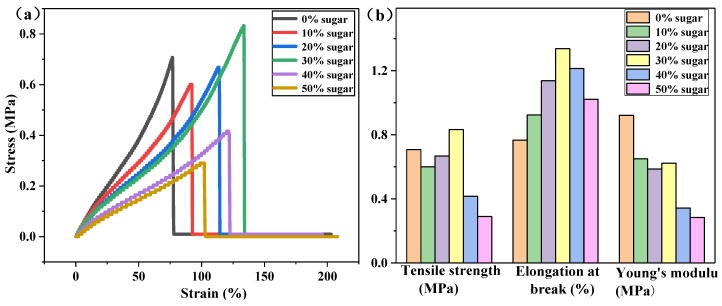
The stress-strain curve and mechanical properties of the sensors with different mass fraction ratios: (**a**) the stress-strain curve of the sensor; (**b**) the mechanical performance diagram of the sensor.

The mechanical properties of the six groups of flexible strain sensor samples with mass fraction ratios of *m*(Sugar)/*m*(PDMS) = *n* (*n* = 0%, 10%, 20%, 30%, 40% or 50%) were evaluated by tensile and compression tests. Figure 4 shows the stress-strain curve and mechanical properties of the six groups of samples. The results of Figure 4a indicate that the porous structure created by sugar dissolution can change the elastic modulus of the material and increase the tensile length of the matrix, thus improving the tensile performance of the sensor. When the mass fraction ratio is 30 wt%, the sensor exhibits the highest variation in tensile strain of up to approximately 130%. Detailed mechanical properties are shown in Figure 4b, where it is evident that at a mass fraction ratio of 30 wt%, the corresponding PDMS substrate has the highest tensile strength and elongation at the breaking point. Therefore, in the subsequent experiments, the PDMS substrate with a mass fraction ratio of 30 wt% was selected as the experimental sample.

The sensitivity (GF) formula of the strain sensor is as follows:

(1) $$GF=(\Delta R/R_{0})/\varepsilon$$

where ΔR is the resistance variation under strain ($\Delta R=R-R_{0}$), $R_{0}$ is the initial resistance without strain, and ε is the relative variation in the length of the sensor under strain. 

**Figure 5:**
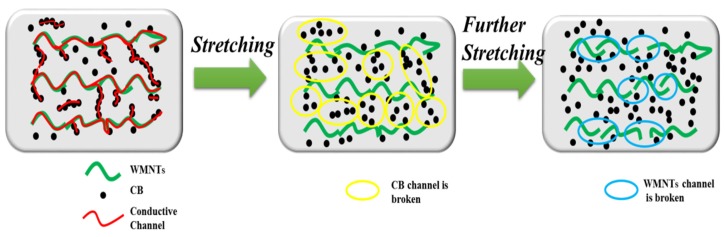
Schematic diagram of the conductive network path of the flexible strain sensor and evolution of the conductive network path under stretching.

The changes in sensor resistance during the strain process are shown in Figure 5. When the sensor is not stretched, no cracks appear on the substrate surface, and there is a close connection between CB-CB, CB-MWNTS, and MWNTS-MWNTS, which forms a large number of conductive pathways (the red curves show conductive pathways in Figure 5). Subsequently, when the sensor is subjected to a small tension force, the substrate exhibits inconspicuous surface cracks, which can be described as larger gaps between carbon black particles in the microscopic structure. Although the separation between CB particles (the yellow circles in Figure 5) reduces the conductive pathways, the connections between CB-MWNTS and MWNTS-MWNTS are affected minimally, and the variation in resistance is thus small. Furthermore, at larger tension forces, obvious cracks appear on the surface of the substrate and the gaps have been widened, resulting in a large number of fractures within the conductive pathways between CB and MWNTS, and between MWNTS (the blue circles in Figure 5). As such, the change in resistance of the sensor increases significantly.

**Figure 6:**
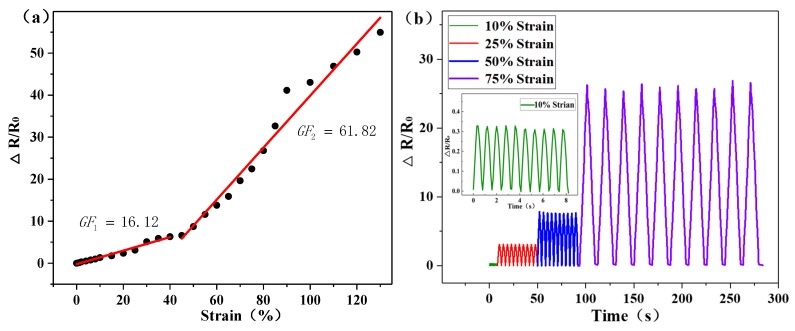

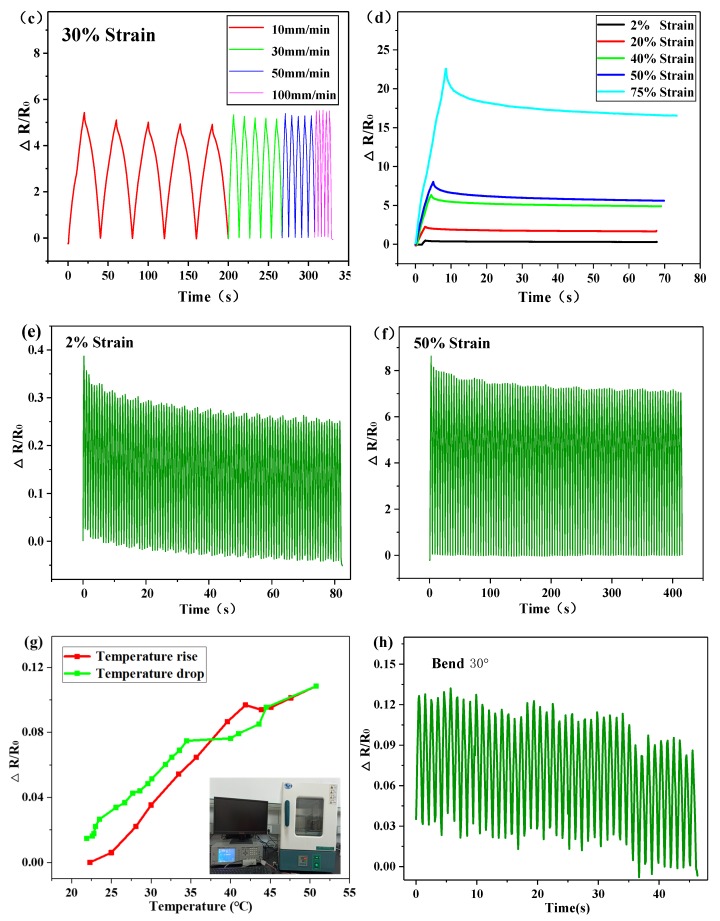
Performance tests of the flexible strain sensor: (**a**) changes in resistance with variations in strain; (**b**) multi-cycle testing of the sensor at different strain levels (10%, 25%, 50% and 75%); (**c**) sensing performance of the sensor at different strain rates at a strain of 30%; (**d**) relative resistance variation for step strain; (**e**) relative resistance variation curve of the sensor for 100 consecutive cycles at a strain of 2%; (**f**) relative resistance variation curve of the sensor for 100 consecutive cycles at a strain of 50%; (**g**) relative resistance variation of the sensor at varying temperatures; (**h**) the relative change in resistance in a cyclic bending test.

The relationship between the variation in resistance $\Delta R/R_{0}$ and tensile elongation (0%–130%) of the sample is shown in Figure 6a. The variation in resistance $\Delta R/R_{0}$ of the strain sensor demonstrates evident monotonous increases as the tensile length is varied. According to the trends of the curve, the GF of the sensor can be divided into two stages: at tensile strains ranging from 0% to 40%, the GF is 16.12, while higher levels of tensile strain between 40% and 130% are characterized by a GF of 61.82. These values show that the strain sensor can be adapted to the different tension ranges. In order to verify the dynamic characteristics of the strain sensor, the sensor was stretched by 2%, 25%, 50% and 75% of its own length, and 10 stretch cycles were performed. The results shown in Figure 6b reveal that the $\Delta R/R_{0}$ changes with the periodic changes in tension force and the sensor has a good regularity and stability. The sensor was held under tensile strain for 70 s in order to verify the stability of the sensor under a prolonged state of tension. Furthermore, Figure 6c shows that the $\Delta R/R_{0}$ tends to be stable after an overshoot peak (overshoot recovery time: 2 s, 5 s, 10 s, 14 s and 17 s), suggesting that the sensor recovers quickly and is able to perform reliably following the overshoot. This overshoot may be due to the acceleration caused by the tension meter as the sensor is stretched. At the same time, the influence of the tensile rate on the strain sensor was also investigated and the results are presented in Figure 6d. The test results show that at a tensile strain of 30%, as the tensile rate increases incrementally from 10mm/min to 100mm/min, the tensile rate has no obvious effect on the $\Delta R/R_{0}$ of the strain sensor. This indicates that under external stresses at different frequencies, the sensor remains stable and can meet the needs of motion detection. The tests were repeated 100 times by loading and unloading the tension variation on the sensor, which was 2% and 50%, to study the robustness and reliability of the sensor under continuous strain. The $\Delta R/R_{0}$ of the sensor under repeated loading at two different levels of tension is shown in Figure 6e,f. The changes observed for the resistance wave form are similar to those of cyclic loading. At the end of the test, the waveform did not show obvious attenuation, which suggests that the sensor maintained an excellent reliability under the action of a repeated strain force. Figure 6g shows that the sensor resistance increases with increasing temperature and decreases as the temperature is reduced. This is due to the thermal expansion of the conductive composite layer with the increase of temperature, and the distance between the conductive fillers increases, resulting in the increase of resistance. In contrast, when the temperature decreases, the conductive composite layer shrinks and the distance between the conductive fillers decreases, resulting in a reduction in resistance. As the temperature increases from 22 to 51 °C, the $\Delta R/R_{0}$ does not exceed 10%, which basically meets the needs of high-temperature conditions.

**Figure 7:**
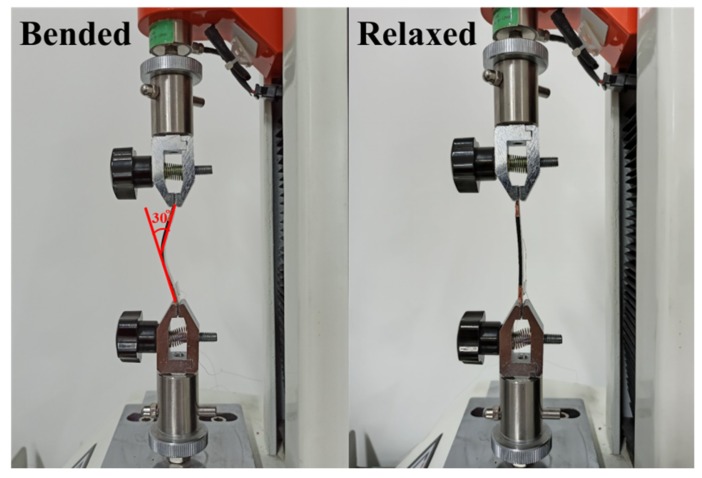
Bending test diagram of the flexible strain sensor on a PET substrate.

Finally, the bending performance of the strain sensor was investigated by attaching the sensor to a PET substrate, which was when installed on the fixture of the bending test instrument in Figure 7. The cyclic compression test was conducted by setting the procedure loop shown in Figure 6h, with the angle of bending set to 30° for 45° repetitions. From the test results below, it can be seen that the resistance of the strain sensor increases during bending, and is restored to its original value after it is released. This illustrates the resilience of the sensor under repeated bending and can meet the needs of bending measurements.

### 3.2. Application of Strain Sensors as Wearable Strain Sensors for Human Motion Detection 

**Figure 8:**
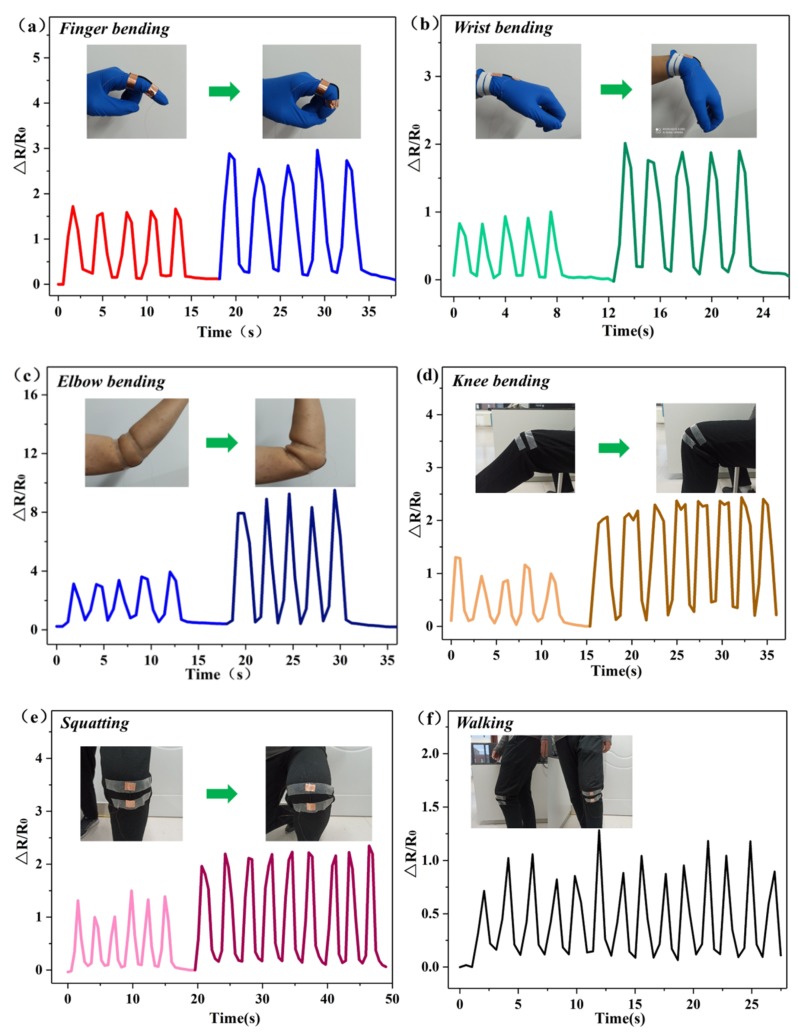

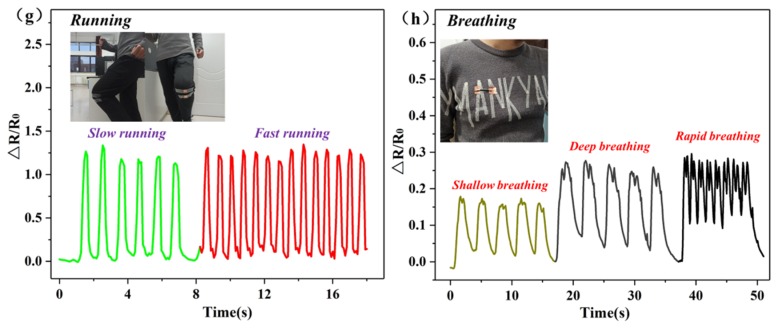
Monitoring of human motions using flexible strain sensors: the responses to human motions of (**a**) finger bending, (**b**) wrist bending, (**c**) elbow bending, (**d**) knee bending, (**e**) squatting, (**f**) walking, and (**g**) running, and (**h**) the responses to breathing.

At present, wearable strain sensors have broad potential for applications in human body information detection due to their ability to respond to external mechanical stimulation. To verify that the sensor presented in this study can indeed be used as a wearable strain sensor, the sensor was attached to different parts of the body for testing. First, the sensors were attached to different joints of the body, and monitored the joint movements of the body in real time, according to the resistance variation. Figure 8a shows a flexible strain sensor on the index finger joint. As the finger joint periodically bends and extends at angles of 45° and 90°, similar periodic changes in $\Delta R/R_{0}$ can be detected. When the finger joint is bent, the strain sensor is in the tensile state, and the $\Delta R/R_{0}$ increases. Similarly, when the finger joint returns to its initial state, the sensor reverts to its original position, and the $\Delta R/R_{0}$ subsequently decreases. Generally, the greater the degree of bending, the greater the deformation of the sensor and the greater the $\Delta R/R_{0}$. As is shown in Figure 8b,c, the strain sensor was also attached to the wrist and elbow joints of the body. It can be observed that when the wrist joint (Figure 8b) and elbow joints bend at angles of 45° and 90° (Figure 8c), the sensor reacts quickly, demonstrating that the strain sensor is sensitive to apparent strain and has a stable repeatability. Furthermore, the sensor was then attached to the knee to provide feedback on human movement monitoring. As shown in Figure 8d, the greater the bending degree, the greater the deformation of the sensor and the greater the $\Delta R/R_{0}$ increases with the bending angle of the knee when the knee is repeatedly flexed at 45° and 90°. Additionally, Figure 8e shows repeated changes in $\Delta R/R_{0}$ during multiple half squats and full squats. The results of knee joint movement detection recorded while volunteers were walking and running are presented in Figure 8f,g. Figure 8g’s results show that the $\Delta R/R_{0}$ changes with the cycle of walking steps, and the frequency of the waveform changes in line with human walking habits. Figure 8h shows the change trend of the $\Delta R/R_{0}$ during the jogging and fast running of volunteers. We can see that the frequency of waveform change during fast running is significantly higher than that during jogging, which is consistent with the actual running situation. This indicates that the sensor can detect the running state. The experimental findings above suggest that the sensor can detect the state of running and can be used to monitor human joint movement.

In addition, sensors were attached to the volunteers’ chests to monitor the $\Delta R/R_{0}$ changes corresponding to shallow, deep, and rapid breathing. As shown in Figure 8h, the $\Delta R/R_{0}$ varies with the respiratory rate as the volunteer breathes. The graph indicates that the $\Delta R/R_{0}$ can change with the rate of respiration when the volunteer is breathing. Compared to shallow breathing, when the human body exhibits deep breathing, the $\Delta R/R_{0}$ displays a greater change. This is because deep breathing increases the strength of the body’s breathing and the expansion of the chest, leading to an increase in strain sensor responses. The $\Delta R/R_{0}$ changes more frequently when the body breathes rapidly compared to normal breathing. At this time, the respiratory rate becomes stronger, causing the strain frequency of the strain sensor to increase. The test result is consistent with the actual situation, which also provides evidence for the abilities of the sensor to detect the physiological activities of the human body in the future.

## 4. Conclusions

In summary, by combining the conductive properties of CB and MWNTS with the high tensile properties of the porous structure of the PDMS substrate, a strain sensor with a unique structure was designed and developed in this study. The experimental results provide evidence which suggests that the porous structure can improve the tensile properties of the sensor, allowing the substrate to be stretched by up to 130%. As CB and MWNTS combine to form a unique nano-composite conductive network structure, the sensor exhibits excellent characteristics, such as a high sensitivity, good linear response, and wide tensile range, as well as good reliability and repeatability. We successfully verified the feasibility of the sensor in terms of motion detection through various experiments. The experimental results show that the stress sensor designed using this kind of method not only performs desirably in terms of movement detection by deformation (such as the movement of fingers, wrists, elbows, knee joints, etc.), but is also capable of detecting the physiological activities of the body (such as the respiration intensity and frequency). Additionally, the simplicity, low cost, manufacturability, and easy integration of the sensor, prove that this design can provide a new feasible approach for motion detection in the field of wearable devices.

## Figures and Tables

**Table 1:** Comparisons of the performances of flexible strain sensing devices.

Reference	Creation Method	Conductive Material	Gauge Factor	Linear Range	Characteristics
[33]	Solution mixture	CB MWNTS	0%–100%-0.91 100%–255%-3.25 255%–300%-13.1	0%–300%	Insensitivity to small strain, inadequate comfort
[38]	Layer-by-layer assembly	CB MWNTS	Maximum to 45.4	15%–150%	Conduction synergy inefficiency
This work	Spraying	CB MWNTS	0%–40%-16.12 40%–130%-61.82	0%–130%	Sensitivity to small strain, high sensitivity
