# Correction: Zhang, P., et al. A Flexible Strain Sensor Based on the Porous Structure of a Carbon Black/Carbon Nanotube Conducting Network for Human Motion Detection. *Sensors* 2020, *20*, 1154

**DOI:** 10.3390/s20102901

**Published:** 2020-05-20

**Authors:** Peng Zhang, Yucheng Chen, Yuxia Li, Yao Zhang, Jian Zhang, Liangsong Huang

**Affiliations:** Key Laboratory for Robot Intelligent Technology of Shandong Province, Shandong University of Science and Technology, Qingdao 266590, China; pengzhang@sdust.edu.cn (P.Z.); chenyucheng@sdust.edu.cn (Y.C.); yuxiali2004@sdust.edu.cn (Y.L.); zhangyao@sdust.edu.cn (Y.Z.); zhangjian@sdust.edu.cn (J.Z.)

The authors wish to make the following corrections to this paper [1]:

There are two non-academic errors should be corrected, we promise that the content of the paper will not be changed as a result of this corrections.

On page 6 of the paper [1], the original wording was as follows: “In order to verify the dynamic characteristics of the strain sensor, the sensor was stretched by 2%, 25%, 50% and 75% of its own length, and 10 stretch cycles were performed. The results shown in Figure 6b reveal that the *R*/*R*_0_ changes with the periodic changes in tension force and the sensor has a good regularity and stability”.

The corresponding Figure 6b in the original paper discusses the dynamic performance test of the sensor when it is stretched to 2%, 25%, 50% and 75% of its own length. Due to a clerical error in drawing, we wrote 10% instead of 2%, and the correct description is 2% instead of 10% which is shown in the following figure.

On page 7 of the paper [1], the original in the article read as follows: “The sensor was held under tensile strain for 70 s in order to verify the stability of the sensor under a prolonged state of tension. Furthermore, Figure 6c shows that the *R*/*R*_0_ tends to be stable after an overshoot peak (overshoot recovery time: 2 s, 5 s, 10 s, 14 s and 17 s), suggesting that the sensor recovers quickly and is able to perform reliably following the overshoot. This overshoot may be due to the acceleration caused by the tension meter as the sensor is stretched. At the same time, the influence of the tensile rate on the strain sensor was also investigated and the results are presented in Figure 6d. The test results show that at a tensile strain of 30%, as the tensile rate increases incrementally from 10 mm/min to 100 mm/min, the tensile rate has no obvious effect on the *R*/*R*_0_ of the strain sensor. This indicates that under external stresses at different frequencies, the sensor remains stable and can meet the needs of motion detection”.

In this paper, the phenomenon described in Figure 6c is actually the phenomenon expressed in Figure 6d in the original text, the phenomenon described in Figure 6d is actually the phenomenon expressed in Figure 6c in the original text. Therefore, the positions of Figure 6c,d need to be interchanged in the paper which is shown in the following figure.

The authors would like to apologize for any inconvenience caused to the readers by these changes in the paper.

## Figures and Tables

**Figure 6:**
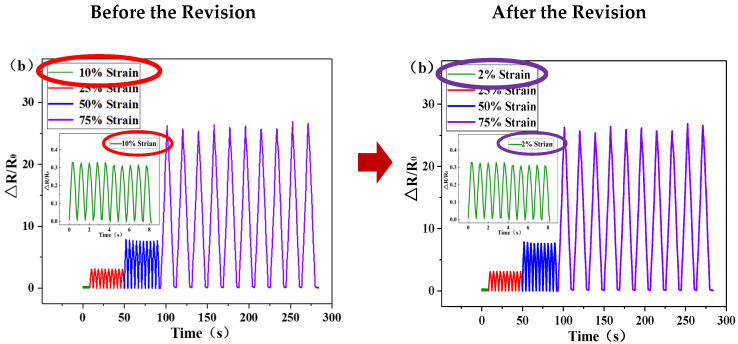
Schematic modification of the first error.

**Figure 6:**
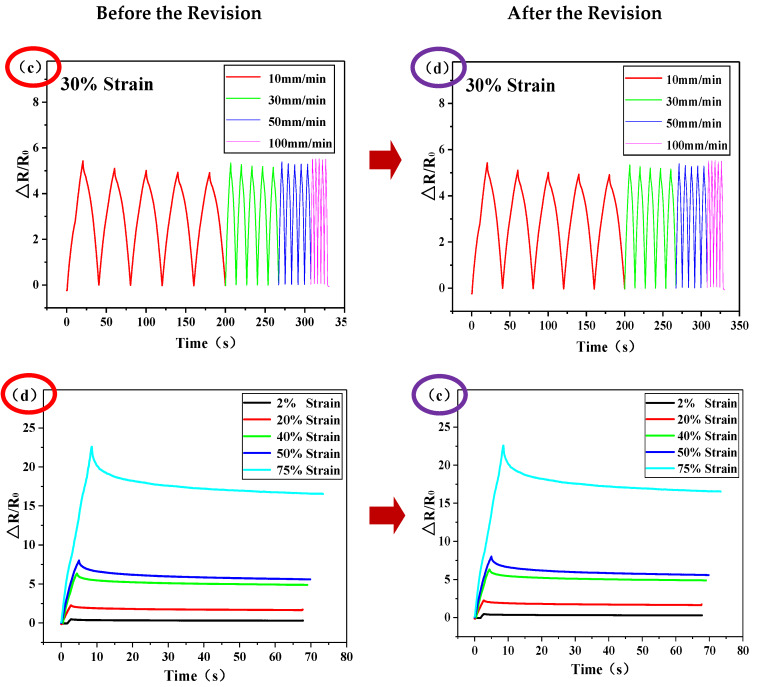
Schematic modification of the second error.
